# Transformative Insights: Break Up with Salt (BUWS) Program’s Short-Term Influence on Hypertension Risk Factors

**DOI:** 10.3390/ijerph21030309

**Published:** 2024-03-07

**Authors:** Jean Pierre Enriquez, Helena Salgado, Christopher Kuetsinya, Li-Hsiang Lin, Elizabeth Gollub

**Affiliations:** 1 School of Nutrition and Food Sciences, Louisiana State University, Baton Rouge, LA 70803, USA; jenriq5@lsu.edu; 2 College of Applied Sciences, University of Illinois, Champaign, IL 61801, USA; helena4@illinois.edu; 3 Department of Mathematics and Statistics, Bowling Green State University, Bowling Green, OH 43403, USA; ckuets@bgsu.edu; 4 Department of Mathematics and Statistics, Georgia State University, Atlanta, GA 30303, USA; lhlin@gsu.edu; 5 School of Nutrition and Food Sciences, Louisiana State University Agricultural Center, Baton Rouge, LA 70803, USA

**Keywords:** hypertension management, community-based nutrition education, DASH diet, food behaviors

## Abstract

Break Up with Salt (BUWS) is a four-session community-based nutrition education program aimed at reducing key controllable hypertension risk factors. This pilot study utilized a pre-post survey design to assess short-term outcomes on food behaviors (including DASH diet eating patterns), physical activity, and overall well-being, in two groups of participants. The first “pilot” group (n = 25) completed a comprehensive, 16-item survey; the second “abbreviated” group (n = 27) completed a 5-item survey. The pilot group experienced improvements in whole grain (*p* = 0.04), sweetened beverage consumption, watching/reducing sodium (*p* = 0.04) and fat (*p* = 0.05) consumption, and time spent sitting (*p* = 0.04). The abbreviated group improved confidence in using food labels (*p* = 0.02), following the DASH diet (*p* < 0.01), preparing food without salt (*p* = 0.03), selecting lower sodium items when eating out (*p* = 0.04), and making a positive lifestyle change (*p* = 0.01). The BUWS program provides information and teaches strategies to manage or prevent hypertension. By effectively improving diet and food behaviors, BUWS has the potential to reduce hypertension risk factors and improve the general health of participants.

## 1. Introduction

Hypertension affects over 33% of our global adult population, over 1.28 billion people worldwide [1]. Hypertension, which is diagnosed when blood pressure is consistently measured at or above 130/80 mmHg, is a primary risk factor for heart disease and stroke and contributes to renal disease, premature death, and disability [2]. Poor diet, dietary sodium, stress, and a sedentary lifestyle are modifiable factors with direct links to this condition [3]. Therefore, some of the most effective non-pharmacological methods for the treatment and prevention of hypertension focus on diet—including sodium restriction, physical activity, stress management, and weight control [3,4,5]. These lifestyle factors are addressed in the Break Up with Salt (BUWS) community-based nutrition education program, a new and innovative curriculum, oriented towards adults, and centered around the DASH diet.

The Dietary Approaches to Stop Hypertension (DASH) diet is recognized for effectively helping to prevent, manage, and treat hypertension [6,7,8,9] and to reduce the risk of stroke, heart failure, and heart disease [10]. The DASH diet was developed in the early 1990s. The blood pressure-lowering effect of this diet was first published in 1997 [11] and is currently considered a principle non-pharmacologic treatment for hypertension [12,13]. As an eating plan, the DASH diet emphasizes vegetables, fruits, whole grains, lean meats/poultry/fish, low-fat dairy products, nuts, seeds, and legumes, monounsaturated and polyunsaturated oils, limited sweets, and no more than 2300 mg sodium/day. The DASH diet also encourages minimally processed and fresh food. DASH is a long-term approach to routine healthy eating. In conjunction with physical activity, stress management, and other healthy lifestyle habits, the DASH eating pattern can help mitigate hypertension, reduce the risk of multiple chronic diseases, and promote general well-being [14].

To adopt the DASH diet, a person must be able to select and prepare foods that align with the DASH principles [15]. For example, the Nutrition Facts label is in place to help consumers make informed choices [16]; those who utilize this label have been found to purchase healthier food products [17]. Helping consumers understand nutrition label information is central to helping them select food items that support their health objectives [18].

Learning or improving food preparation and cooking skills are equally critical for maintaining DASH eating patterns. Lack of cooking knowledge, confidence, and skills tends to limit at-home meal preparation [19], yet meals obtained outside the home contain more fat, sodium, sugar, and energy [20,21] and less micronutrients and fiber [22]. Among adults, cooking skills have been directly correlated with greater vegetable consumption and healthier food preparation methods [23]. In general, culinary skills empower individuals to plan and prepare flavorful healthy meals that include a variety of foods [24].

Dietary management of high blood pressure can be enhanced with routine physical activity and stress management practices [4,25]; simple techniques can be used to integrate and routinize these practices. Daily exercise of any amount has a positive effect on individuals with high blood pressure [26], and routine physical activity will help prevent age-associated increases in blood pressure [27]. Daily exercise supports weight maintenance and helps to cope with stress, both of which are effective means of controlling blood pressure. Stress and perceived stress have a direct influence on the autonomic nervous system and arterial pressure [28], yet physical activity helps to mitigate the negative effects and to maintain blood pressure within normal parameters [26]. It has also been noted that individuals who participate in moderate to intense physical activity are more likely to have higher DASH diet compliance scores [29]. Given the relationships among diet, physical activity, stress, and hypertension, each of these lifestyle components plays an important role in hypertension management [25].

Hypertension management is fundamental to health promotion and health equity. This is particularly relevant to Louisiana where the adult hypertension rate (~40%) is among the highest in the United States [30]. BUWS was developed by nutrition and extension experts at the LSU AgCenter. The curriculum focuses on the DASH diet, food selection, and cooking methods, while also integrating goal-setting and habit-formation techniques, and guidance on physical activity and stress management. The purpose of this pilot study was to evaluate the short-term influence of BUWS program participation on food selection, preparation, and consumption behaviors; on engagement in physical activity; and on stress, sleep, and health.

## 2. Methods

### 2.1. Program/Evaluation Design

BUWS conveys the logic and potential of key lifestyle choices in the management or prevention of hypertension. The program was designed to convey practical knowledge and develop skills among its participants to reduce key controllable risk factors. The BUWS pilot was conducted virtually from September 2020 to March 2021. The pilot participant evaluation was designed to track changes in diet, physical activity, and sitting behavior, as well as stress, health, and sleep quality. A pre–post survey design was integrated into the program to capture short-term change. Surveys were distributed and submitted electronically. Encouraged by the findings of the pilot study, BUWS was implemented, virtually or in-person, from January to December of 2022 with an abbreviated, 5-item participant evaluation survey. This study was approved (exempted) by the Institutional Review Board (IRBAG-20-0013).

### 2.2. Program Description

The BUWS curriculum consists of a series of four class sessions to be conducted weekly, over a 4-week period, by nutrition extension agents trained on the delivery of the BUWS content and on the BUWS evaluation component. Each of the four classes focuses on a different topic (Table 1). The virtual program was developed in the summer of 2020, during the COVID-19 pandemic. An alternative, content-comparable in-person version has been available since January 2022. Virtual sessions were not to exceed 1 h; in-person sessions could be 60–90 min. Both session formats include demonstrations, hands-on learning opportunities, and support/reference materials.

### 2.3. Agent Training

The nutrition agent training was conducted online over two consecutive days, for a 2-hour period each day. The first day covered curriculum content, i.e., objectives, scripts, audience engagement activities (e.g., food demos), and support materials (e.g., informational handouts) for each of the four sessions. The second day focused on the operational aspects of the program. These included the use of the technology, program promotion, session scheduling/organization, participant registration, and facilitation of the participant evaluation protocol (including participant consent).

### 2.4. Participants

The BUWS program targeted adults with hypertension or who have a family member with hypertension. However, registration was open to any interested adult. The BUWS pilot evaluation study participants were adults (>18 years old) living in Louisiana, registered for the BUWS program, and who agreed to participate in the evaluation process (not required for program participation). Nutrition agents around the state promoted the program locally through newsletters, newspapers, social media, and email lists. BUWS was marketed by word-of-mouth to health professionals and other community contacts.

### 2.5. Participant Survey Instrument

The BUWS pilot participant evaluation survey, both pre- and post-program, consisted of 16 core items covering foods/eating patterns associated with the DASH diet, physical activity/inactivity, food/product labels, food selection, and perceived stress and health (Table 2). Most of the questions were adopted from the Behavior Risk Factor Surveillance System [31], the National Health and Nutrition Examination Survey [32], or the STEPwise approach to surveillance [33]. Some measures were adapted from validated tools such as the Food Skills and Cooking Skills Confidence Measures [34] and the Perceived Stress Scale [35]. The survey structure was based on a tool previously developed for use with similar populations [21].

### 2.6. Survey Testing

The BUWS participant survey was pre-tested using a cognitive interview process (mid-August 2020), typically used to identify question–response issues with survey items [36]. Five participants reflecting the anticipated age, race, and educational diversity of program participants, were recruited for this purpose. An item-by-item review with each pre-tester informed minor changes. Overall, it was determined that each item was understandable and interpreted by the participant as it was intended to be.

### 2.7. Abbreviated Survey

After the pilot data were analyzed and facilitating agents were debriefed, it was decided that the core participant survey, pre and post, would be abbreviated to 5 items. This was to reduce the participant reporting burden and encourage evaluation participation while continuing to capture data from the key reporting categories of the original participant evaluation survey. The items still covered, though in more general terms, indicators of foods/eating patterns associated with the DASH diet, use of salt, and small changes in lifestyle behaviors to improve blood pressure (Table 3). The abbreviated participant survey was used with both virtual and in-person programs.

### 2.8. Data Analysis

Participants from the pilot evaluation and the 2022 evaluation were characterized by gender, race, and age range using descriptive statistics. A strength of the inferential analysis is that it took the timeline and sample size into consideration.

#### 2.8.1. The Pilot Evaluation

The BUWS pilot evaluation study involved a restricted participant pool in which not all participants completed both pre- and post-program surveys. This resulted in a dataset where certain data pairs were matched, while other data remained unmatched. This scenario is recognized in statistical terms as “partially matched data”. To effectively analyze these data, particularly for assessing changes from pre-program to post-program periods, the Wilcoxon test for partially matched data was employed [37]. This non-parametric test conducts a rank-based analysis on two-sample data. It is particularly robust as it does not rely on specific parametric distribution assumptions. This is achieved by integrating both matched and unmatched datasets for a comprehensive evaluation. This test is suitable for ordinal, Likert, and numerical response types by testing for direction of change, increasing or decreasing [38]. Pre- and post-responses were used to determine change in behavior, confidence, or perception. The variables evaluated include sodium reduction, fat reduction, physical activity, sitting time, stress, ability to handle stress, use of ingredients and substitutions, food skills, and use of nutritional labels (Table 2). All data were analyzed using a one-tailed hypothesis method at a 5% significance level; the one-sided testing direction is identified with the survey results. All data were analyzed with a 95% confidence level.

#### 2.8.2. The 2022 Evaluation

The 2022 abbreviated survey data analysis used the Wilcoxon signed-rank test, which can detect whether the proportion of responses increased or decreased from the pre- to post-program period [37]. This statistical test accommodates the Likert response elicited from each of the abbreviated survey’s 5 core items (Table 3). As with the pilot data, a one-tailed hypothesis method at a 5% significance level and 95% confidence level, was used for analysis; the testing direction is identified for each survey item.

## 3. Results

The BUWS pilot was implemented in 13 of the state’s 65 parishes, representing rural (45%) and urban (55%) communities from each region of the state. The 2022 evaluation involved participants from five parishes, also representing each region of the state.

In both the pilot and 2022 evaluations, the majority of participants (>88%) were female. Pilot participants were exclusively female; the 2022 evaluation included three male participants (Table 4). More than half of the participants (52%) from the pilot evaluation were 51–64 years of age. Participants in the 2022 evaluation tended to be older, with approximately 82% as 65+ years. In both evaluations, the participant group ranged from approximately 45–48% White and 42–48% Black or African American.

The pilot program recruited 25 evaluation participants, all of whom completed the pre-program survey. However, only 18 completed the post-program survey. In 2022, 27 participants registered and completed the pre-program survey, but only 17 completed the post-program survey.

### 3.1. The Pilot

Among the group of pilot participants, whole grains was the only DASH diet food (or beverage) consumption category with significant pre–post change. However, the participants reported a desirable direction of change in the amount or type of fat consumed and in sodium intake over the 4-week program period (Table 5). Although there was no change in time spent on physical activity, there was a significant decrease in time spent sitting (Table 5). Changes in the amount of stress, ability to handle stress, sleep quality, and quality of health were not detectable during this program period, nor were they expected to be. These core items were intended as a baseline for longer-term follow-up.

Among the items reflecting skills and behaviors associated with food acquisition or preparation (items 10–16), only several presented a significant change (Table 6). The participants began to ask more questions about food ingredients and substitutions when eating away from home, they increased the practice of packing their own meals or snacks for times they are away from home, and they increased their use of the Nutrition Facts label when shopping/decision-making (Table 6). There was the appearance but no statistically significant improvement in confidence in food preparation or selection skills.

### 3.2. The 2022 Evaluation

Analysis of responses to the five abbreviated evaluation items indicated a statistically significant (*p* < 0.05) increase in each one. After participating in the four BUWS sessions, there was more statistical evidence to support positive food/lifestyle behaviors (Table 7).

## 4. Discussion

The BUWS program presents information and teaches skills and strategies to help manage or prevent hypertension. The program centers on the DASH diet and related food/lifestyle behaviors. This short-term evaluation study utilized self-reported pre–post changes in performance or confidence in selecting, preparing, and consuming food. Engagement in physical activity and sedentary behavior were also considered. Participation in the original pilot and in the 2022 evaluation was modest but sufficient in number and representation to demonstrate, given the methodology, that the BUWS program has a positive influence on participants.

Over the study period, the pilot participants reported increased consumption of whole grains and decreased consumption of sugar-sweetened beverages, both of which can impact blood pressure [39]. Grains, especially bread, can be perceived as an unfavorable food choice [40]. However, during the BUWS program, participants appeared to have begun to associate the consumption of whole grains and unsweetened beverages with healthier choices. Similarly, BUWS participation appears to have positively influenced dietary fat and sodium, aligning with DASH [6,7,8,9,14] and a healthier eating pattern in general [39]. BUWS session discussions covered healthy fats such as oils and nuts, prior to which participants might not have understood or acknowledged as “healthy” fats [41].

Sessions also highlighted the relationship between sodium and hypertension, the sodium content of food products, and options for preparing flavorful food without salt. BUWS participants appear to have learned these lessons as noted by the post-program increases in confidence and behaviors associated with sodium reduction. Other researchers have demonstrated that a DASH-based nutrition education hypertension management program can reduce sodium consumption and blood pressure [42], such that as a strategy, DASH diet interventions are recognized as effective [43]. BUWS included a discussion on the value of potassium-rich vegetables, fruits, and legumes for blood pressure management. Among the BUWS participants, evidence of significant improvement in consumption of vegetables, fruits, processed meats, nuts and seeds, and legumes categories might have been more subtle, requiring a longer period for detection.

Improvements in physical activity, stress, or the ability to handle stress were anticipated but not ascertained. However, there was a significant decrease in the amount of time the pilot participants spent sitting, which has been directly associated with hypertension [44]. Because sitting, physical activity, and stress are closely related [45], it is possible that over time, physical activity will increase and stress will decrease among these participants [46]. Physical activity and stress are shown to influence the quality of sleep [47] and overall health [48]. The BUWS pilot participants did not report changes in sleep or health, but improvements remain a reasonable, longer-term expectation.

In the U.S., the frequency of consuming meals prepared outside the home has been increasing over the decades [49]. At an average of approximately 1300 mg sodium per meal, this accounts for approximately 25% of the dietary sodium of U.S. adults [50]. After the four BUWS sessions, the pilot participants became more confident about checking ingredients and/or requesting the substitution of a meal item when eating away from home. Applied to sodium and fats, as well as to vegetable or grain selections, this dietary strategy could improve diet quality over time. In general, after the four BUWS sessions, the participants reported increased confidence in cooking skills and healthy food preparation at home. Interestingly, confidence in knife skills among the pilot participants was down. It is possible that these participants experienced temporary uncertainties as they learned “correct” techniques.

Food label literacy is considered a critical component of nutrition education, with applications to grocery stores, prepared food venues, and the home [51]. Those who use food labels while food shopping tend to identify, purchase, and consume healthier choices [52]. This appears to be the case among the BUWS participants. As a group, the pilot and 2022 evaluation participants increased confidence in their ability to read labels and identify healthy foods in general. Among the pilot participants, improvements in label use were also reported when shopping specifically for salad dressings and canned/frozen vegetables—both potentially high-sodium products—and when selecting potentially highly sweetened beverages. The ability to read and use the Nutrition Label likely supported decreased consumption of sodium and sweetened beverages and the decreased and/or modified fat consumption reported by the participants.

Beyond the label, food selection is influenced by internal and external factors such as sensory perception, access, and knowledge, as well as personal motivation, routine, or experience [53]. The BUWS evaluation participants were sufficiently motivated by their health concerns to attend program sessions and complete the evaluation process. Such motivation tends to result in behavior changes [54]. Still, the BUWS program attempted to integrate and utilize all these influences in each program session to encourage progressive adoption of DASH diet food behaviors.

BUWS was developed as a community education program to help adults prevent or manage hypertension. The strength of the BUWS program content lies in its comprehensive approach to targeted dietary and lifestyle behaviors. BUWS teaches techniques that impact blood pressure by encouraging the selection, preparation, and consumption of a diet rich in potassium, calcium, and fiber and low in sodium, saturated fats, and added sugars, as well as a more physically active lifestyle. The strength of this BUWS evaluation study lies in its assessment of food/diet, physical activity, and sitting behaviors or confidence to perform behaviors that represent these key controllable hypertension risk factors. The primary limitations of this study include the modest participation rates and the study length. This study was based on the strong influence of the DASH diet, physical activity, and sedentary behavior on blood pressure. This study utilized these factors as a proxy for blood pressure management, which could be considered a limitation or a strength.

## 5. Conclusions

In the short term, the BUWS evaluation participants reported improvements in key behaviors that help to manage hypertension over the long term. The participants also demonstrated increased confidence in skills associated with healthy food behaviors suggesting that the development of additional positive food behaviors will manifest over time. As a community nutrition education program focused on hypertension management, BUWS has the potential to improve diet and general health among participants.

## Figures and Tables

**Table 1:** Topics and descriptions from the BUWS lessons.

Lesson	Topic/Session Title	The Focus of the Session
1	Understanding Hypertension; How to Form Heart-Healthy Habits	An overview of hypertension, of controllable vs. uncontrollable risk factors, and of techniques for making small, sustainable lifestyle changes to help lower (and maintain lower) blood pressure.
2	Understanding the DASH Diet to Help Manage High Blood Pressure	A description of the DASH diet and how to adapt a DASH diet pattern as a routine eating style. The Nutrition Facts label and how to use nutrition label information to select foods for better blood pressure management.
3	Dash in for Groceries	A guided, virtual grocery store tour to practice label reading, mitigating food product misunderstandings, and selecting healthier foods; foods that align with the DASH diet.
4	Mastering Meals with Flavor	Preparation methods and use of seasonings to create flavorful but lower sodium dishes and meals.

**Table 2:** Pilot 1 participant survey—core items.

Item No.	Survey Question	Response Type
1.	Yesterday how many times did you eat: (a) vegetables; (b) fruits; (c) whole grains; (d) processed meats; (e) nuts or seeds; (f) legumes.	numerical
2.	Yesterday how many times did you drink: sweetened beverages	numerical
3.	Are you currently watching or reducing your fat intake or changing the type of fat you use?	nominal
4.	Are you currently watching or reducing your sodium or salt intake?	nominal
5.	In a typical week, on how many days do you do any type of physical activity that causes an increase in breathing or heart rate? And on average, how much time/day?	numerical
6.	On a typical day, how much time do you usually spend sitting?	numerical
7.	During the past 30 days, how would you rate: (a) the amount of stress in your life; (b) your ability to handle stress?	Likert
8.	During past 3 months how would you rate your quality of sleep?	Likert
9.	During the past 3 months, how would you rate your health?	Likert
10.	When you eat foods prepared away from home, do you ask about the ingredients or possible substitutions or alternative seasoning?	ordinal
11.	Typically, do you prepare main meals at home, using herbs and spices for seasoning?	ordinal
12.	On days that you go to work, school, take road trips or are just out and about/away from home, do you prepare and pack your own meals or snacks?	ordinal
13.	How confident are you in your ability to: (a) Slice, dice, mince, chop, and peel vegetables, herbs, and spices? (b) Select and use herbs and spices for your food? (c) Prepare healthy meals at home?	Likert
14.	How confident are you in your ability to: (a) Determine which foods are the healthier options when selecting foods from restaurants, food stands, fast food outlets, vending machines etc.? (b) Distinguish between “every day” foods and “occasional” foods?	Likert
15.	How often do you use the Nutrition Facts label when deciding to buy a food product?	ordinal
16.	How often do you look for nutrition information on the Nutrition Facts label when you buy each of the following types of foods: (a) Snacks; (b) Breakfast cereals; (c) Salad dressings/fats/oils; (d) packaged seasonings; (e) Pre-packaged foods; (f) Canned or frozen vegetables; (g) Breads, tortillas, prepackaged grain products; (h) Milk, yogurt, cheese, ice cream; (i) Juices, fruit drinks, teas, soda, waters?	ordinal

**Table 3:** Abbreviated participant survey—core items.

Item No.	Question: Using a Scale of 1 to 4 Where 1 Is the Lowest and 4 is the Highest, Please Mark How Confident You Are That You Know How to:
1	Use food labels when grocery shopping, to figure out which foods are healthier for you.
2	Select foods and fix meals that follow the DASH eating plan.
3	Prepare food that tastes good without adding salt.
4	Make lower sodium food choices when eating out (e.g., restaurants, food stands, convenience stores, fast food, vending, etc.).
5	Make small, simple lifestyle changes to help manage your blood pressure.

**Table 4:** Demographics of program evaluations.

	Pilot Evaluation ^1^		2022 Evaluation	
	n	%	n	%
Gender				
Male	0	0.0	3	11.1
Female	24	96.0	24	88.9
Prefer not to say	1	4.0	0	0.0
Race				
White	11	45.8	13	48.2
Black or African American	10	41.7	13	48.2
Hispanic or Latino	1	4.2	0	0.0
Other/skipped	2	8.3	1	3.7
Age				
35 to 50	5	20.0	1	3.7
51 to 64	13	52.0	4	14.8
65 or over	7	28.0	22	81.5

^1^ All pilot participants reported being food secure (“enough of the kinds of food we want to eat” or “enough but not always the kinds of food we want to eat”). Among the group of pilot participants, 13 resided in rural areas and 12 in urban neighborhoods.

**Table 5:** Pre-to-post participant change in selected pilot evaluation measures.

Survey Item	Pre-Survey Mean (n = 25)	Post-Survey Mean (n = 18)	Direction of Change ^1^	*p*-Value ^2^
1(c)—Whole grains	1.1	1.3	Increase	0.04
2—Sweetened beverages	1.4	1.2	Decrease	0.01
3—Watching/reducing fat or changing type of fat	1.6	1.8	Increase	0.05
4—Watching/reducing sodium intake	1.5	1.9	Increase	0.04
5—Hour per week of physical activity	1.6	1.4	Increase	0.07
6—Sitting time (hours) per day	6.0	5.0	Decrease	0.04
7(a)—Amount of stress	3.2	3.6	Decrease	0.60
7(b)—Ability to handle stress	2.4	2.9	Increase	0.44
8—Quality of sleep	2.6	2.7	Increase	0.87
9—Quality of health	2.7	3.0	Increase	0.91

^1^ Wilcoxon test for partially matched data direction as tested (increase or decrease). ^2^ *p* < 0.05.

**Table 6:** Food skills/behaviors: ingredients and substitutions, confidence in food skills, use of Nutrition Facts label.

Survey Item	Pre-Survey Mean (n = 25)	Post-Survey Mean (n = 18)	Direction of Change ^1^	*p*-Value ^2^
10—Ask about ingredients or substitutions when eating away from home	1.4	2.1	Increase	0.01
11—Herbs and spices in place of salt for seasoning	2.4	2.7	Increase	0.90
12—Prepare and pack meals/snacks for when away from home	2.0	3.5	Increase	<0.01
15—Use Nutrition Facts label [in general]	3.3	3.8	Increase	0.02
16(c)—Salad dressing	3.0	3.3	Increase	0.05
16(f)—Canned/frozen vegetables	2.5	3.8	Increase	0.01
16(i)—Juices, fruit drinks, teas, soda, and waters	2.7	3.2	Increase	0.05
Confidence in ability to:				
13(a)—Slice, dice, mince, chop, and peel vegetables, herbs, and spices	4.6	4.2	Increase	0.46
13(b)—Select and use herbs and spices for your food	3.3	3.9	Increase	0.43
13(c)—Prepare healthy meals at home	3.4	3.9	Increase	0.11
14(a)—Determine which foods are the healthier options when selecting foods from restaurants, food stands, fast food outlets, vending machines, etc.	3.3	4.1	Increase	0.16
14(b)—Distinguish between “every day” foods and “occasional” foods	3.6	3.8	Increase	0.43

^1^ Wilcoxon test for partially matched data direction as tested (increase). ^2^ *p* < 0.05.

**Table 7:** Pre-to-post participant change—2022 evaluation survey items.

Improved Confidence in Knowing How to:	Pre-Survey Mean (n = 27)	Post-Survey Mean (n = 17)	Direction of Change ^1^	*p*-Value ^2^
1—Use of food labels for grocery shopping, to identify healthy foods	2.7	3.4	Increase	0.02
2—Select foods and fix meals that follow the DASH diet	1.7	3.1	Increase	<0.01
3—Prepare food that tastes good without adding salt	2.5	3.1	Increase	0.03
4—Make lower sodium food choices when eating out	2.5	2.9	Increase	0.04
5—Make small, simple lifestyle changes to help manage blood pressure	2.8	3.4	Increase	0.01

^1^ Wilcoxon sign rank test for proportion change direction as tested (increase). ^2^ *p* < 0.05.

## Data Availability

The data could be made available through the corresponding author.
